# Measurable Residual Disease (MRD) Monitoring in Myeloproliferative Neoplasms

**DOI:** 10.3390/cancers18203397

**Published:** 2026-10-20

**Authors:** Gary E. Markey, Stephen E. Langabeer

**Affiliations:** Cancer Molecular Diagnostics, St. James’s Hospital, 8 Dublin Dublin, Ireland; slangabeer@stjames.ie

**Keywords:** myeloproliferative neoplasm, *JAK2*, *CALR*, *MPL*, NGS, ddPCR, polycythemia vera, essential thrombocythemia, primary myelofibrosis

## Abstract

*BCR::ABL1* negative myeloproliferative neoplasms (MPN) are driven predominantly by somatic mutations in *JAK2*, *CALR*, and *MPL*, which underpin diagnosis, prognostication, and measurable residual disease (MRD) monitoring. Advances in molecular assays have improved sensitivity for detecting canonical drivers and additional mutations across the MPN spectrum. In this review we evaluate current and emerging molecular methodologies for polycythemia vera (PV), essential thrombocythemia (ET), and primary myelofibrosis (PMF). We review the utility of PCR-based approaches, including allele-specific PCR, real-time quantitative PCR, and droplet digital PCR (ddPCR). Advances in ddPCR demonstrate precision and lower limits of detection for *JAK2* V617F, *CALR*, and non-driver mutations, thus supporting the expanding role of ddPCR in MRD monitoring and relapse prediction. Recent advances in NGS techniques provide targeted profiling, enabling identification of possible co-occurring driver mutations and additional mutations associated with disease progression, treatment resistance, and leukemic transformation. Collectively, emerging workflows complement established gold-standard methodologies, while offering enhanced analytical performance and deeper pathological insight across the MPN spectrum. Optimal method selection requires balancing assay sensitivity, cost, and clinical utility. Advances in ddPCR and NGS methodologies have applicability for MRD monitoring in MPN and are powerful tools for detection of heterogenous and co-occurrent mutations across the MPN spectrum.

## Simple Summary

The classical *BCR::ABL1* negative myeloproliferative neoplasms (MPN) are predominantly driven by mutations in three genes: *JAK2*, *CALR*, and *MPL*. Detection and quantitation of these mutations is important for disease diagnosis and monitoring. This review explores molecular methods used to monitor measurable residual disease (MRD) in polycythemia vera (PV), essential thrombocythemia (ET), and primary myelofibrosis (PMF) by tracking these mutations. Polymerase chain reaction (PCR)-based tests have been widely adopted, while emerging techniques like droplet digital PCR (ddPCR) can detect extremely low levels of mutations with high accuracy. Next-generation sequencing (NGS) provides a broader insight into genetic changes, helping identify additional mutations linked to disease progression or treatment resistance. Improvements in sensitivity are likely to further the use of NGS approaches for MRD monitoring. These evolving methods will allow for greater scope for disease monitoring in the setting of established and novel emerging therapeutic modalities.

## 1. Introduction

The *BCR::ABL1* negative myeloproliferative neoplasms (MPN) are a group of hematopoietic stem cell malignancies characterized by discordant hematopoietic stem cell proliferation resulting in clonal expansion in one or more myeloid lineages [1,2]. These classical *BCR::ABL1* negative MPNs are polycythemia vera (PV), essential thrombocythemia (ET), and primary myelofibrosis (PMF) and have a combined prevalence of approximately 100–150 cases per 100,000 persons worldwide. Annual incidence rates are estimated at 0.84, 1.03, and 0.47 cases per 100,000 persons for PV, ET, and PMF, respectively [3]. Although PV, ET, and PMF share common molecular origins through constitutive activation of the JAK-STAT signaling pathway, they differ in their clinical manifestations. PV is characterized by erythrocytosis, with patients commonly presenting with headache, dizziness, pruritus, erythromelalgia, thrombotic events, and splenomegaly. ET is distinguished by sustained thrombocytosis and is often asymptomatic, although patients may experience headache, thrombosis, or hemorrhage. PMF has the most aggressive phenotype and is characterized by bone marrow fibrosis, with symptoms including fatigue, weight loss, night sweats, fever, progressive anemia, and often marked splenomegaly due to extramedullary hematopoiesis [4,5,6].

PV and ET possess the potential to extend to a myelofibrotic phenotype, as well as with PMF to progress to acute myeloid leukemia (AML) [1,4,5]. MPN are driven by acquired gain-of-function somatic mutations, namely affecting *JAK2* (*JAK2* V617F, *JAK2* exon 12), *CALR*, and *MPL* genes resulting in the constitutive activation of the JAK-STAT signaling pathway [6,7,8,9]. The *JAK2* V617F mutation occurs in >95% of PV diagnoses and >50% in ET and PMF [2,6], while ~3% of PV patients in whom the *JAK2* V617F is not detected harbor a mutation in *JAK2* exon 12 [9]. Moreover, mutations in exon 9 of the *CALR* gene occur in ~30% of ET and PMF cases [10,11]. Among the range of mutations found within *CALR* exon 9 the most common is a 52bp deletion (Type 1), followed by a 5bp insertion (Type 2), with type-1 and type-2-like mutations occurring less frequently [12,13]. The least common driver mutations are in the *MPL* gene and occur in 5–10% of ET and PMF patients [14,15]. The molecular mechanisms underlying aberrant signaling in MPNs, have been extensively reviewed elsewhere. Readers are referred to the following reviews for a more comprehensive overview [16,17]. The acquisition of driver mutations was once considered mutually exclusive, however advances in next-generation sequencing (NGS) workflows have shown that this is not the case, with some patients displaying mutations in more than one driver gene, the co-occurrence of second driver mutations was shown more commonly in patients with low variant allele frequency (VAF) *JAK2* V617F mutations [18,19,20]. The absence of these classical driver mutations does not negate an MPN diagnosis, as these patients are referred to as triple-negative patients and often display additional mutations indicative of a *BCR::ABL1*-negative MPN.

The utility of molecular profiling is a mainstay for diagnosis and measurable residual disease (MRD) monitoring. MRD is the persistence of detectable clone-driving mutations below the threshold of standard clinical detection, which may reflect response to treatment and identification of relapse [4,5]. Identification of canonical driver mutations allows for comprehensive diagnosis that may serve as targets for MRD monitoring. Unlike acute leukemias, MRD monitoring is not routinely indicated for all MPN patients because disease-associated driver mutations frequently remain detectable despite clinical and hematologic response. MRD monitoring is most clinically relevant following therapies such as interferon treatment and allogenic stem cell transplantation (ASCT) [21,22].

Emerging capabilities in droplet digital PCR (ddPCR) and NGS workflows have seen adoption of these methods in molecular diagnostics of MPN with indications for disease stratification and MRD. While considerable attention has been devoted to the analytical performance of MRD assays, the clinical relevance of MRD monitoring is equally important. The value of MRD monitoring includes the potential to provide prognostic information, evaluate treatment response, and guide clinical decision-making. In MPNs, recurrent driver mutations such as *JAK2*, *CALR*, and *MPL* provide molecular markers that can be quantified over time and used to assess residual disease burden [22]. Herein we provide an overview of molecular methodologies with a comparison to current approaches and the utility for MRD monitoring with respect to the *BCR::ABL1* negative MPN.

## 2. Techniques for Monitoring MRD in MPN

Advances in NGS, RQ-PCR, and ddPCR have seen improved assay sensitivity, particularly in the utility of diagnosis, prognosis, and MRD monitoring (Table 1). The ICC and WHO recommend the use of single-target PCR or multitarget NGS assays with a sensitivity <1% to support a diagnosis of PV, ET or PMF [4,5]. While multiparameter flow cytometry is an established modality for MRD assessment in acute leukemias and other hematological malignancies due to leukemia-associated phenotypes [23,24], its application in classical *BCR::ABL1*-negative MPNs is limited by the absence of immunophenotypic markers and the maturity of the peripheral blood cells. Consequently, MRD monitoring in PV, ET, and PMF relies predominantly on molecular detection of somatic mutations using highly sensitive PCR and sequencing-based methodologies, flow cytometry is not considered a dedicated MRD methodology. MPN driver mutations are valuable diagnostic targets as well as occasionally providing utility in monitoring disease response, as seen in post-ASCT [25,26]. Although the role of these driver mutations in clinical evaluation of emerging therapies has not been fully elucidated.

### 2.1. JAK2

JAK2 is a non-receptor tyrosine kinase involved in erythropoiesis and thrombopoiesis and controls adequate cell proliferation and differentiation. *JAK2* mutations perturb signaling and lead to constitutive STAT activation, thus potentiating uncontrolled proliferation. Allele-specific PCR is a popular assay for the detection of *JAK2* V617F mutation [27,28]; however, with the advances in high-throughput NGS and ddPCR, these latter technologies are becoming increasingly popular in detecting variants, and supporting the diagnosis of MPN in the separation of triple-negative cases and for possible MRD monitoring [4,22]. Initial inter-laboratory comparisons showed the utility of RQ-PCR for the detection and MRD monitoring for *JAK2* V617F disease burden [29], with recent advances in ddPCR providing improved sensitivity and quantitation of disease burden across the MPN spectrum [30,31] (Table 1). Research on the clinical utility of MRD monitoring is shown in studies of JAK inhibitor therapy. Although ruxolitinib and other JAK inhibitors reduce splenomegaly and improve constitutional symptoms, studies have shown that these agents can also decrease the *JAK2* V617F mutant allele burden. In a long-term follow-up analysis of patients receiving ruxolitinib, reductions in *JAK2* V617F allele burden were observed over time, with a subset of patients achieving partial or complete molecular responses. Importantly, decreases in allele burden correlated with reductions in spleen volume, suggesting that molecular responses may reflect meaningful clinical improvement. This observation supports the potential use of serial molecular monitoring as an adjunct measure of treatment response [32].

Several studies have shown the comparison of qPCR and ddPCR methodologies for the detection of *JAK2* V617F in MPN. One study compared a cohort of 99 MPN patients with a *JAK2* V617F mutation by qPCR and ddPCR, showing ddPCR possessed higher sensitivity compared to qPCR, as well as demonstrating an elevated allelic burden from ET to PV and PMF [33]. A separate study confirmed the improved sensitivity of ddPCR. In 63 MPN patients, six treated with ruxolitinib were comparatively tested by qPCR and ddPCR. It was found that both PCR methodologies displayed good sensitivity and could detect *JAK2* V617F mutations, with ddPCR having an improved limit of detection of 0.01% compared to 0.12% for qPCR. Authors demonstrated the advantage of utilizing ddPCR in MRD monitoring, especially when allele burden is below the limit of detection for routine qPCR assays [34]. The clinical utility of ddPCR for *JAK2* V617F detection was further investigated whereby 59 MPN patients, some of whom had undergone ASCT, were subject to qPCR and ddPCR. Authors showed a limit of detection of 0.01% for both assays and detected *JAK2* V617F in 43 out of 59 patients using both PCR methodologies. Interestingly, ddPCR detected *JAK2* V617F mutations in 14% of the samples from patients having recently undergone allogenic ASCT, thus demonstrating the improved sensitivity of ddPCR in the identification of a low *JAK2* V617F allele burden [35].

Another study compared the technical performance of qPCR and ddPCR in a cohort of 195 *JAK2* V617F-positive patients. The authors showed that ddPCR displayed optimal accuracy, reproducibility, and higher sensitivity in the quantification of *JAK2* V617F allele burden compared to qPCR. Authors showed that ddPCR has improved precision for the detection of lower frequency mutations, with improved utility of ddPCR in MRD monitoring [31]. The progression of PMF has been associated with an increasing *JAK2* V617F allele burden. Studies have demonstrated a variation in allele burden across different MPN phenotypes. The lowest allele burden was associated with ET, and a higher allele burden was observed in PV and PMF cases [36,37,38]. Moreover, a separate study showed the application of ddPCR-based MRD monitoring post-ASCT in *JAK2* V617F-positive MF patients and described it as an accurate tool for the prediction of relapse and post-ASCT survival [26,39]. In contrast, the application of RQ-PCR techniques offers good sensitivity and is capable of detecting low allelic burden consistent with *JAK2* exon 12 mutations [40,41]. Although, it is not the preferred diagnostic method due to the heterogeneity of mutations within this region.

NGS detects *JAK2* V617F mutations with comparable performance to that of routine PCR-based methodologies, as well as the capability to detect potentially new *JAK2* variants, albeit with less robust sensitivity than conventional PCR-based assays [42]. Although High Resolution Melting Analysis offers high sensitivity and robust detection of *JAK2* exon 12 mutations at low allelic frequency [43,44]. Since the initial sequencing of exon 12 [8], there has been considerable understanding of the range of mutations within this region, with more than 60 unique mutations identified [45]. In this regard, sequencing is the gold standard for detecting *JAK2* exon 12 mutations due to the frequency and diversity of these mutations. Whereby initial standardization of *JAK2* detection via PCR-based methodologies provided robust data for MRD [29,46,47,48], these approaches could perhaps be translated into the emerging methodologies of ddPCR and NGS. This would allow the potential application of ddPCR and NGS in gauging treatment response across the MPN spectrum.

### 2.2. CALR

The JAK/STAT pathway is activated in patients with *CALR* mutations through interaction with MPL, with *CALR* exon 9 mutations representing the second most frequent driver mutations of MPN. *CALR* mutant patients display lower leukocyte counts, lower hemoglobin levels, higher platelet counts, and a lower thrombosis risk. Methodologies for the detection of *CALR* mutations, which are generally restricted to patients with ET and PMF, must be capable of detecting the abundance of indels that currently exist. Among these, fragment analysis PCR is the most common and has displayed effectiveness for MRD monitoring [49,50]. Previous work has shown the retrospective use of fragment analysis in a cohort of PMF patients post-ASCT mirrored donor chimerism status in relapsing patients, thus demonstrating the utility of the method for monitoring of *CALR* mutation burden [51].

A comparison of the detection limits of a number of molecular techniques using cell lines with a 61bp deletion reported detection limits between 10–25%, 5%, 5%, and 1.2 % for Sanger sequencing, fragment analysis, high-resolution melt analysis, and NGS, respectively [52]. Although fragment analysis is the most rapid and cost-effective for diagnostic screening, it possesses a relatively high limit of detection and remains semi-quantitative. Similarly, with a sensitivity of 10–20%, Sanger sequencing is deemed unsuitable for MRD (Table 1). In contrast, NGS displays high sensitivity but is constrained by high cost and turnaround time. PCR assays offer high sensitivity and affordability, are more broadly available for a wide range of molecular targets, and have been used in *CALR* type-1 and type-2 mutations with a detection limit that did not exceed 1%, thus remaining suboptimal for deep MRD monitoring [53]. In contrast, ddPCR allows for the accurate quantification of allele burden and may be suitable for MRD [26,54,55] (Table 1). One study developed a ddPCR assay for the detection of type-1 and 2 *CALR* allelic burden and was found to be more reproducible and accurate than common fluorescent PCR assays. Furthermore, in patients with PMF post-ASCT this assay was able to predict relapse in accordance with the reappearance of *CALR* mutations [56]. Further studies have developed ddPCR assays for the quantification of *CALR* type-1 and type-2 mutations with applicability for MRD monitoring [57,58]. Collectively, these data suggest the advantages of ddPCR for *CALR* mutation quantification and continual assessment for MRD monitoring [45,59].

### 2.3. MPL

Most MPN-associated *MPL* mutations are located within exon 10 and affect tryptophan residue W515. W515L and W515K are the most common, with others (W515A, W515S, W515R) having been described [60]. Several methodologies have been developed for the detection of *MPL* mutations, including Allele-Specific Oligonucleotide PCR, High-Resolution Melting analysis, and Allele-Specific PCR, which have varying levels of sensitivity ranging from 1.5 to 5% [61,62,63]. Furthermore, RQ-PCR in conjunction with High-Resolution Melting analysis or the utility of bidirectional Sanger sequencing has been shown to have increased sensitivity of 0.2% [64] (Table 1). The difficulty in the implementation of PCR methodologies is dependent on the distribution of mutations as well as the low mutational frequency, which has limited the use of *MPL* as an MRD marker. Previous studies have shown the utility of qPCR in identifying clearance of MRD in PMF patients post ASCT [65,66].

**Table 1:** Comparative assay sensitivities for canonical MPN driver mutations.

	RQ-PCR	ddPCR	AS-PCR	HRMA	Sanger	NGS	Reference
*JAK2* V617F sensitivity	0.01–1%	0.008–1%	0.001–1%	0.5–10%	10–20%	1–2%	[22,31,34,35,42,67]
*JAK2* exon 12 sensitivity	~2%	NA	0.05–1%	5–10%	10–20%	1–2%	[22,43,44,45,68]
*CALR* sensitivity	1–2%	0.02–0.1%	<4%	2.5–5%	10–20%	1–2%	[22,26,45,52,53,54,57]
*MPL* sensitivity	0.2–2%	NA	0.1–3%	2–5%	10–20%	1–2%	[22,45,61,62,63,64]

### 2.4. Triple-Negative MPN

*JAK2*, *CALR*, and *MPL* are present in 90% of MPN cases. However, the absence of these drivers is seen in approximately 15% of ET and 10% of PMF patients [7,16,69]. Research comparing the molecular features of 254 PMF patients with driver and non-driver mutations (*ASXL1*, *EZH2*, *IDH*, *SRSF2*, *SF3B1*, and *U2AF1*) via PCR and bidirectional sequencing showed that triple negative patients displayed lower leukemia-free survival [69]. Moreover, the utility of ddPCR as a sensitive and reliable technique for the quantification of non-driver allelic burden has been evaluated for diagnostic reliability. One study looked at the application of ddPCR, in this regard, for *IDH1*, *IDH2*, and *DNMT3A* in 162 PMF patients post-ASCT with authors showing that among the 162 screened patients, 13 had *IDH* and *DNMT3A* mutations, as well as concomitant driver mutations (*JAK2* and *CALR*). Data confirmed that ddPCR was sufficient for the detection of non-driver mutations in the identification of relapse and was concordant when normalized to pre-transplant allele frequencies [70]. Overall, triple-negative PMF patients are associated with poorer survival and elevated risk of leukemic transformation, with the application of ddPCR allowing for identification and quantification of allelic burden of alternative mutations for MRD monitoring.

### 2.5. NGS Applications for MRD

With the capacity of NGS having a central role in diagnostic and prognostic workflows in hemato-oncology, the availability of these methods for MRD monitoring has been put to the forefront. The application of NGS pipelines in AML to identify mutations and monitor MRD post-treatment lends itself to the MPNs. Firstly, one study utilized an applicable single-molecule molecular inversion probe (smMIP)-based NGS MRD assay targeting 92 loci in 33 AML driver genes in 93 AML patients. The authors showed this assay provided robust data for patient risk-stratification pre-ASCT, identified potential treatment-induced mutagenesis, and was cost-effective with low library preparation costs [71]. Moreover, recent advances in NGS methodologies have challenged previous recommendations surrounding the utility of *FLT3*-ITD as an MRD marker in AML. The ELN-DAVID MRD working group applied ultra-high sensitivity NGS and yielded improved identification and quantification of *FLT3*-ITD mutations, thus allowing for MRD monitoring of patient-specific *FLT3*-ITD clones using specialized bioinformatic pipelines [72]. The utility of NGS techniques is that they are powerful tools in assessing MRD due to multi-gene analysis capabilities. One study designed an NGS-MRD pipeline in AML and included *JAK2*, *MPL*, and *CALR* as part of the panel. Although the presence of these drivers is often low in *de novo* AML, the inclusion of these drivers aids in MPN diagnosis but additionally has value in monitoring MRD of secondary AML arising from MPN [73].

Similarly, the application of NGS for the detection of additional gene mutations within key regulatory pathways surrounding transcriptional regulation and cell signaling has been used to predict the risk of leukemic transformation and may have applicability for MRD (Table 2). The strongest clinical indication for MRD monitoring in MPNs is post-ASCT, where early detection of molecular relapse can facilitate timely therapeutic intervention. Post-transplant molecular monitoring enables early detection of potential relapse, often before overt recurrence. Current EBMT/ELN recommendations support regular monitoring of driver mutations at 30, 100, 180, 270, and 360 days post-ASCT to facilitate early interventions, such as donor lymphocyte infusion. Studies have highlighted the prognostic significance of combining molecular monitoring with chimerism analysis to improve relapse detection and post-transplant risk stratification [21,74].

A key consideration for MRD monitoring in *BCR::ABL1*-negative MPNs is the stability of the MRD marker itself. Although canonical driver mutations (*JAK2*, *CALR*, and *MPL*) are generally stable throughout the disease and represent suitable MRD targets, clonal evolution is a recognized feature of MPN progression. Acquisition of additional mutations in genes involved in epigenetic regulation, RNA splicing, and signaling pathways contributes to disease progression, therapeutic resistance, and leukemic transformation (Table 2). Importantly, transformation of MPN to secondary AML may occur from a clone that is distinct from the dominant chronic-phase clone, resulting in apparent loss of the original driver mutation. Thus, reliance on a single driver mutation for MRD monitoring may underestimate disease burden or fail to detect emerging high-risk mutations. This limitation highlights the potential value of NGS-based methodologies, which permit simultaneous monitoring of driver and non-driver mutations and may provide a more comprehensive assessment of clonal burden over time. Therefore, the clinical utility of MRD monitoring depends not only on analytical sensitivity but also on the biological stability of the selected molecular marker throughout the disease course [27,28,69,75].

Data acquired through the use of NGS, which defined additional mutations between PV and ET patients, is correlated with survival data and risk of transformation. From a treatment perspective, several studies have shown the association of adverse mutations and treatment intolerance. According to one study, resistance to hydroxyurea has been estimated in 64% of patients with *TP53* mutations and 49% in patients with spliceosome or chromatin gene mutations [76] (Table 2). Another study investigated patients with ET and *CALR* mutations following IFNα treatment and showed that attenuation of treatment was associated with mutations in *TET2*, *IDH2*, *ASXL1*, and *TP53* [77]. Separately, the presence of *DNMT3A* mutations has also been associated with IFNα resistance and failure to acquire a complete molecular response [78].

Among patients with PMF, it is more common to observe additional mutations than patients with other MPNs, the majority of which are associated with methylation, chromatin remodeling, splicing, and DNA repair mechanisms. Mutations in *ASXL1*, *EZH2*, *IDH1/2*, and *SRSF2* have been associated with poor survival and elevated risk of transformation to AML [28,79]. Similarly, a comparative study to understand the number of additional mutations, impact on overall survival, and leukemic-free survival in a cohort of 797 PMF patients confirmed, using NGS, that the presence of two or more high-risk mutations in *ASXL1*, *EZH2*, *SRSF2*, and *IDH1/2* had diminished overall survival and leukemic-free survival, compared to PMF patients with one or no additional mutations [80]. In comparison, another study showed that *ASXL1*, *TET2*, *SRSF2*, *U2AF1*, *CBL*, *KIT*, *RUNX1*, *CEBPA*, and *SH2B3* mutations were the most frequent in PMF patients and were associated with poorer survival [28]. Further insight into the progression of PMF was carried out using a targeted NGS panel of 137 genes, whereby authors analyzed the mutation status of selected genes to better understand the mutational landscape and enrichment of non-driver mutations between pre-fibrotic PMF (Pre-PMF), overt PMF (Overt-PMF), and accelerated/blast phase PMF (PMF-AP/BP). *ASXL1*, *TET2*, *SRSF2*, *U2AF1*, and *SETBP1* were the most commonly mutated. Whereas *ASXL1* and *U2AF1* were more frequent in overt-PMF. Moreover, mutations in *ASXL1*, *SRSF2*, *RUNX1*, *SETBP1*, *NOTCH2*, *NRAS*, and *EZH2* were more elevated in PMF-AP/BP than those in overt-PMF [75]. The use of NGS methods in the stratification of PMF patients is highly valuable for treatment selection and prognostication over disease course. Research has shown that mutations in *RAS/CBL* are associated with resistance to ruxolitinib and poor survival, highlighting the importance of identification of these mutations in patients with PMF [81,82]. Overall, the application of NGS-based assays provides extensive information for patients with PMF, particularly surrounding the scope for treatment response and MRD monitoring (Table 2).

The capacity of modern NGS platforms and bioinformatics pipelines in the context of MPN diagnostics allows for the sequencing of multiple genes of interest and identification of mutations with high specificity and improved sensitivity. Targeted sequencing of canonical driver mutations or implementation of wider-scale NGS panels for additional mutation analysis has MRD capabilities across the MPN spectrum. Although the limitations in the utility of NGS for routine MRD monitoring in MPN may be currently cost prohibitive and have relatively slower turnaround times [11,67,83], direct cost comparisons between methodologies are difficult because published cost data are limited and vary substantially according to laboratory setting, instrumentation, assay design, sample throughput, and regional systems. Further research is required for the standardization and routine clinical applicability of NGS pipelines for MRD monitoring in MPN [71,84,85].

**Table 2:** Most common additional mutations and frequencies in MPN.

	Gene	Frequency			Reference
		PV	ET	PMF	
Driver mutation	*JAK2*	>95%	50–60%	50–60%	[2,4,6,27,28,68,79,80]
	*MPL*	<1%	5–10%	5–10%	[27,28,68,79,80]
	*CALR*	<1%	~30%	~30%	[13,27,28,68,80]
DNA methylation	*TET2*	10–20%	3–10%	10–20%	[13,27,28,79,83,86]
	*DNMT3A*	5–10%	1–5%	5–15%	[13,27,28,70,79,86,87]
	*IDH1/2*	1–2%	1–2%	~5%	[13,27,28,69,70,79,80]
Chromatin modification	*ASXL1*	2–7%	5–10%	15–35%	[13,27,28,68,69,75,79,80,86,87]
	*EZH2*	1–2%	1–2%	7–10%	[13,27,28,69,75,79,80,86]
RNA splicing	*SRSF2*	~2%	~2%	5–14%	[13,27,28,69,75,79,80,87]
	*SF3B1*	2–3%	2–5%	5–7%	[13,27,28,69,83,87]
	*U2AF1*	<2%	<2%	7–10%	[13,27,28,75]
Signaling	*KRAS/NRAS*	<2%	<2%	2–4%	[13,27,28,69,75]
	*CBL*	<2%	<2%	4%	[13,27,28,81,82,86]
	*KIT*	~2%	~2%	<2%	[27,28]
	*SH2B3*	<9%	2–5%	2–5%	[27,28,86]
Transcriptional regulation	*RUNX1*	<2%	<2%	2–3%	[13,27,28,75,87]
	*CEBPA*	2–5%	4%	<10%	[27,28]
	*TP53*	<5%	5–9%	5%	[27,28,76,77,87]

## 3. Conclusions

With advances across molecular methodologies, detection of driver mutations in *BCR::ABL1* negative MPN provided a breakthrough in diagnosis and patient monitoring. The utility of ddPCR and NGS workflows provides scope for continued improvements in sensitivity and dynamic range for MRD assessment of MPN patients. Implementation of MRD workflows requires consideration with regard to patient cohorts, as well as the technical capacities of the chosen MRD assay. The utility and improvement of MRD assays in MPN, particularly with reference to ddPCR and NGS pipelines, requires continued standardization with regard to how these data are reported with prospective proficiency testing to ensure translation into optimal treatment response and disease management. MRD detection is actionable primarily post-ASCT, where molecular relapse often precedes clinical relapse. MRD assessment also has value in evaluating the depth of response. Among available methodologies, ddPCR currently appears to be the most practical tool for clinical decision-making, owing to high sensitivity, relatively low cost, and ability to quantify driver mutations. In contrast, NGS provides broader biological and prognostic information through detection of co-occurring and high-risk mutations, making it valuable for risk stratification and prediction of treatment resistance, yet higher cost, longer turnaround time, and lack of standardization currently limit routine use for MRD-guided decisions; however, optimal method selection requires balancing assay sensitivity, cost, and clinical utility with the methodologies available [26,39].

Combination therapeutics are increasingly emerging as a promising utility to overcome the limitations of some current therapies in MPNs. Although JAK inhibitors and interferon therapy have transformed MPN management through improvements in symptom burden and splenomegaly, the capacity to achieve deep molecular responses remains limited. Therefore, considerable interest has focused on combining current therapies with agents targeting complementary pathogenic mechanisms, including epigenetic dysregulation, inflammatory signaling, and mutant cell persistence. Early studies suggest that these approaches may only enhance clinical responses and promote reductions in driver mutation burden and improvements in bone marrow fibrosis, supporting the possibility of disease modification. As more effective combinations are studied, MRD monitoring would be beneficial in assessing treatment response and detecting residual malignant clones. Ultimately, the convergence of combination therapies and MRD monitoring may facilitate a shift from symptom control towards precision-guided management in patients with MPNs [78,88,89,90,91].

In summary, the advances in molecular assays have improved mutation detection across the continuum of *BCR::ABL1* negative MPN. These methods provide robust and comprehensive data that has been shown across patient diagnosis, monitoring of treatment response, and prediction of relapse and progression. The complex molecular landscape underpinning MPN therefore requires considered assay selection and data interpretation. In light of a host of disease-modifying agents in development [88], optimizing management strategies with the integral inclusion of MRD should be able to provide individualized patient pathways for improved long-term outcomes.

## Data Availability

No new data were created or analyzed in this study. Data sharing is not applicable to this article.

## Abbreviations

The following abbreviations are used in this manuscript:

MPN	Myeloproliferative Neoplasm
PV	Polycythemia Vera
ET	Essential Thrombocythemia
PMF	Primary Myelofibrosis
AML	Acute Myeloid Leukemia
ddPCR	Digital Droplet PCR
NGS	Next Generation Sequencing
AS-PCR	Allele-Specific PCR
RQ-PCR	Real-Time Quantitative PCR
HRMA	High-Resolution Melting Analysis
